# Sex-specific regulation of cardiac microRNAs targeting mitochondrial proteins in pressure overload

**DOI:** 10.1186/s13293-019-0222-1

**Published:** 2019-02-06

**Authors:** Hugo Sanchez-Ruderisch, Ana Maria Queirós, Daniela Fliegner, Claudia Eschen, Georgios Kararigas, Vera Regitz-Zagrosek

**Affiliations:** Charité – Universitätsmedizin Berlin, corporate member of Freie Universität Berlin, Humboldt-Universität zu Berlin, and Berlin Institute of Health, Institute of Gender in Medicine and Center for Cardiovascular Research, and DZHK (German Centre for Cardiovascular Research), partner site Berlin, Germany

**Keywords:** Cardiac, ERβ, microRNA, Mitochondrial, Pressure overload, Sex differences

## Abstract

**Background:**

Maladaptive remodeling in pressure overload (PO)-induced left ventricular hypertrophy (LVH) may lead to heart failure. Major sex differences have been reported in this process. The steroid hormone 17β-estradiol, along with its receptors ERα and ERβ, is thought to be crucial for sex differences and is expected to be protective, but this may not hold true for males. Increasing evidence demonstrates a major role for microRNAs (miRNAs) in PO-induced LVH. However, little is known about the effects of biological sex and ERβ on cardiac miRNA regulation and downstream mitochondrial targets. We aimed at the analysis of proteins involved in mitochondrial metabolism testing the hypothesis that they are the target of sex-specific miRNA regulation.

**Methods:**

We employed the transverse aortic constriction model in mice and assessed the levels of five mitochondrial proteins, i.e., Auh, Crat, Decr1, Hadha, and Ndufs4.

**Results:**

We found a significant decrease of the mitochondrial proteins primarily in the male overloaded heart compared with the corresponding control group. Following computational analysis to identify miRNAs putatively targeting these proteins, our in vitro experiments employing miRNA mimics demonstrated the presence of functional target sites for miRNAs in the 3′-untranslated region of the messenger RNAs coding for these proteins. Next, we assessed the levels of the functionally validated miRNAs under PO and found that their expression was induced only in the male overloaded heart. In contrast, there was no significant effect on miRNA expression in male mice with deficient ERβ.

**Conclusion:**

We put forward that the male-specific induction of miRNAs and corresponding downregulation of downstream protein targets involved in mitochondrial metabolism may contribute to sex-specific remodeling in PO-induced LVH.

## Background

Under pressure overload (PO) conditions as in aortic stenosis or hypertension, left ventricular (LV) hypertrophy (LVH) develops. Following the initial adaptive LV remodeling, when PO persists, maladaptive LV remodeling leads to the development of heart failure. In the process of LVH development and the transition to heart failure, major sex differences have been reported (reviewed in [1]). Briefly, women develop a more concentric form of LVH with less ventricular dilation and wall thinning than men, thereby leading to sex-specific cardiac dysfunction [2, 3]. We and others have reported a stronger induction of fibrosis and fibrotic gene expression in the male versus female overloaded heart [4–6]. Similarly, our previous studies with experimental animals revealed that the female sex is associated with less pronounced ventricular dilation and fibrosis in response to PO [7]. On the basis of the identified sex-specific fibrotic gene regulation, we further reported the sex-specific regulation of six fibrosis-related microRNAs (miRNA) in the mouse overloaded heart [8].

miRNAs are small non-coding RNAs of approximately 22 nucleotides [9] that negatively regulate gene expression pairing to the 3′ untranslated region (3′-UTR) of target messenger RNAs (mRNAs) [10]. The expression of several miRNAs has been described in the mouse heart, many of which are regulated during the development of LVH [11–15]. However, the impact of biological sex on miRNA regulation and the relevance for the documented sex differences in cardiovascular disease are poorly understood.

The steroid hormone 17β-estradiol (E2), along with its receptors ERα and ERβ, is thought to play a major role in the development of sex differences in cardiovascular disease. In fact, the E2/ER axis is expected to be protective, but this may not hold true for males. In females, though, ERβ has been shown to confer cardio-protection under PO [16–18]. The mechanisms underlying these effects have been the subject of intense investigations (reviewed in [1]), and we have shown that deletion of ERβ leads to the modulation of several genes in the overloaded heart [7, 19]. However, it is not clear to what extent mitochondrial proteins might be regulated in a sex-specific manner under PO and what the role of miRNAs might be in this regulation. An aberrant regulation of mitochondrial function and bioenergetics in the heart, a high energy-demanding organ, could be detrimental leading to LV dilation and dysfunction.

On the basis of the aforementioned transcriptomic studies in PO [7, 19], we aimed here at characterizing under PO the modulation of five proteins, namely, AU RNA binding methylglutaconyl-CoA hydratase (Auh), carnitine O-acetyltransferase (Crat), 2,4-dienoyl-CoA reductase 1 (Decr1), hydroxyacyl-CoA dehydrogenase alpha subunit (Hadha), and NADH:ubiquinone oxidoreductase subunit S4 (Ndufs4), due to their importance and involvement in mitochondrial metabolism. We tested the hypothesis that these proteins are the target of sex-specific miRNA regulation.

## Methods

### Experimental animals

Male and female C57Bl/6J mice at the age of 2 months were employed. To study the effects of ERβ on miRNA regulation in vivo, ERβ knockout (ERβ^−/−^) mice generated from heterozygous mouse colonies [20] were employed. The genotype of the mice was screened using PCR amplification as described previously [20]. The mice were kept on a 12–12-h light/dark cycle in temperature-controlled rooms with water ad libitum. All experiments were carried out in accordance with the EU Directive 2010/63/EU for animal experiments and approved by the Landesamt für Gesundheit und Soziales, Berlin, Germany.

### Transverse aortic constriction

To induce PO in mice, the transverse aortic constriction (TAC) method was used as described previously [7]. Two-month-old mice were anesthetized with ketamine hydrochloride (80 mg/ml)/xylazine hydrochloride (12 mg/ml) administered by intraperitoneal injection at a dose of 1 mg/kg. Sham animals underwent an identical surgical procedure without placement of a suture. Animals recovered from anesthesia under warming conditions and normal ventilation. After 9 weeks, the mice were sacrificed by isoflurane overdose followed by laryngotomy. Following weight measurements, the hearts were snap frozen in liquid nitrogen and stored at − 80 °C until further analysis.

### Immunoblotting

Protein lysates were isolated from LV tissue samples as described previously [21, 22]. Independent biological replicates for each group were run separately on SDS-PAGE gels and transferred to nitrocellulose membranes using standard procedures. Primary antibodies against Auh (ab155980, Abcam), Crat (ab153699, Abcam), Decr1 (sc-366484, Santa Cruz), Hadha (sc-82185, Santa Cruz), Ndufs4 (ab139178, Abcam), Tim23 (611222, BD; control for mitochondrial structural integrity), Tom40 (sc-365466, Santa Cruz; control for mitochondrial structural integrity), and tubulin (T9026, Sigma-Aldrich; loading control) were used. For immunodetection, secondary antibody donkey anti-rabbit, anti-goat, or anti-mouse (Dianova) and ECL™ Prime Western Blotting Reagent (Amersham) were used. Data were quantified with the ImageJ 1.41 version software (http://rsbweb.nih.gov/ij/).

### Computational analysis

TargetScan (http://www.targetscan.org) was used for the identification of putative miRNA binding sites in the 3′-UTRs of the mRNAs coding for the mitochondrial targets as previously described [8, 23].

### Cell culture experiments

The cardiac muscle cell line HL-1 was used for target validation studies and cultured as described previously [24, 25]. For luciferase/renilla reporter assays, 3′-UTRs [Auh NM_016709.2, Crat NM_007760.3, Decr1 NM_026172.3, Hadha NM_178878.2, Ndufs4 NM_010887.2] or putative target sites were site directed cloned (XhoI/NotI) in psiCheck™-2 (Promega) behind the renilla coding region and a Dual-Glo® Luciferase Assay (Promega) was performed. Transient transfection was performed with FuGene HD (Promega) or Attractene (Qiagen) following the provider’s instructions [26]. The assays including the addition of specific miRNA mimic (1 μl, 20 μM/well) were done by electroporation using the Amaxa™ Cell Line Nucleofector™ Kit V (Lonza, protocol T020) followed by a second transfection with the reporter clones 24 h later. The following miRNA mimics were used: Syn-mmu-miR-106a-5p, MSY0000385; Syn-mmu-miR-130a-3p, MSY0000141; Syn-mmu-miR-133a-3p, MSY0000145; Syn-mmu-miR-143-3p, MSY0000247; Syn-mmu-miR-19b-3p, MSY0000513; Syn-mmu-miR-199b-5p, MSY0000672; Syn-mmu-miR-23a-3p, MSY0000532; Syn-mmu-miR-24, MSY0000219; Syn-mmu-miR-27b-3p, MSY0000126; Syn-mmu-miR-29a-3p, MSY0000535; Syn-mmu-miR-497a-5p, MSY0003453; Syn-mmu-let-7e-5p, MSY0000524 (Qiagen).

### Real-time quantitative RT-PCR

RNA was isolated using the miRNeasy kit (Qiagen). Purified miRNAs were reverse transcribed with the miScript Reverse Transcription kit (Qiagen), followed by real-time PCR using the QuantiTect SYBR Green PCR kit (Qiagen) using the primers let-7e: TGAGGTAGGAGGTTGTATAGTT; miR-23a: TCACATTGCCAGGGATTT; miR-27b: TCACAGTGGCTAAGTTCTGC; miR-130a: AGTGCAATGTTAAAAGGGC; miR-133a: CCCCTTCAACCAGCTG; miR-TGAGATGAAGCACTGTAGCTC. The amount of miRNA was normalized using the average of the expression of RNU6B and RNU1A (Qiagen internal references).

### Statistical analysis

All data are presented as mean ± SEM. Statistical significance was assessed using the R 2.14.2 software. Comparisons among multiple groups were made with two-way ANOVA and Tukey’s post hoc test adjusting for multiple comparisons. *P* ≤ 0.05 was considered significant.

## Results

### Sex-specific regulation of the mitochondrial targets under pressure overload

**Fig. 1:**
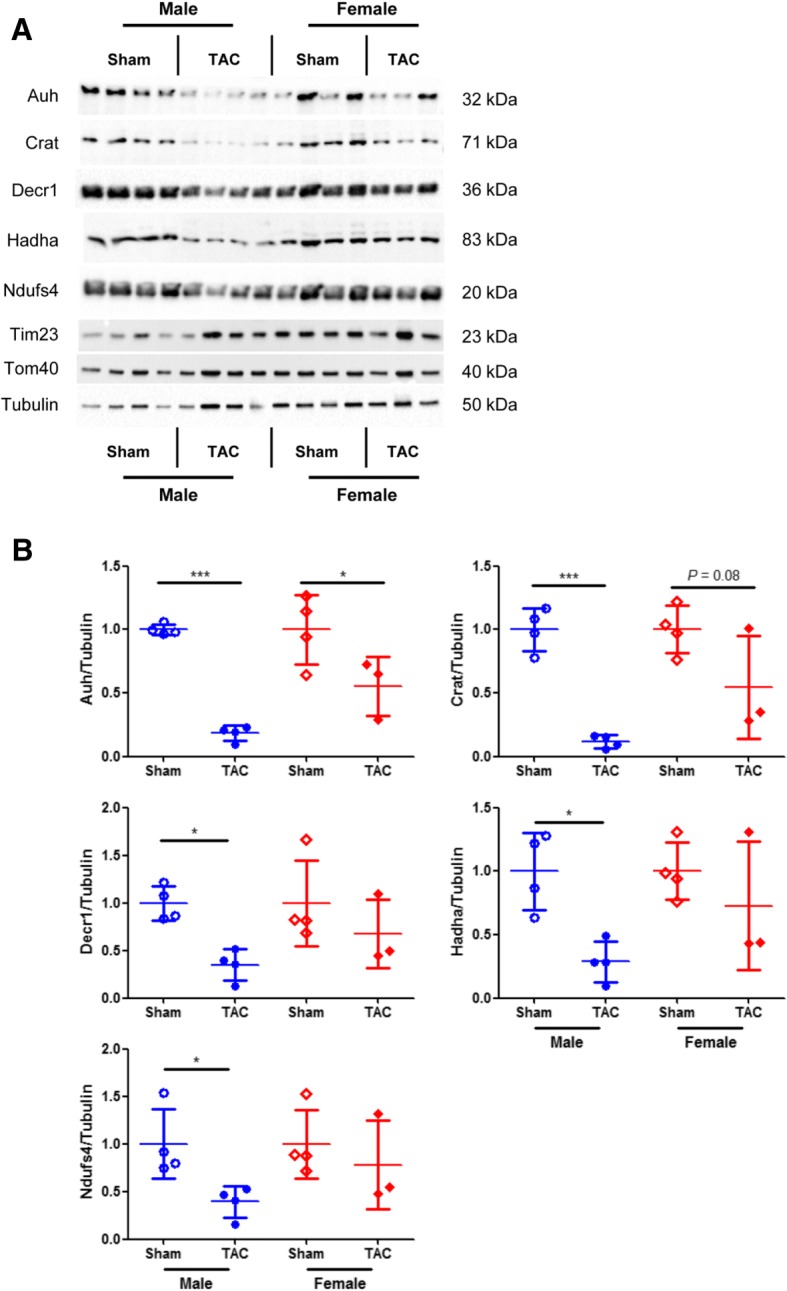
Sex-specific mitochondrial protein regulation under pressure overload. Representative immunoblots (**a**) and quantification of protein abundance (**b**) in LV samples from male sham (*n* = 4), male TAC (*n* = 4), female sham (*n* = 4), and female TAC (*n* = 3) mice. Tim23 and Tom40 were used as controls for mitochondrial structural integrity and tubulin as loading control. Data are presented in scatter dot plots including mean with SD. ***P < 0.001, *P < 0.05

Our previous human and animal studies on PO have indicated the sex-specific regulation of mitochondrial metabolism-related genes [4, 7]. Based on this, among the identified regulated candidates, we selected five proteins, due to their importance and involvement in mitochondrial metabolism, and assessed their levels in response to PO. For this purpose, we analyzed LV samples from male and female mice subjected to PO (TAC vs. sham), which showed significant sex differences in the development of LVH (Table 1). In line and as we had hypothesized, we found that the selected mitochondrial proteins were regulated in a sex-specific manner (Fig. 1). In particular, the abundance of all five proteins was significantly decreased in male mice subjected to TAC compared with the corresponding sham group (Fig. 1). In contrast, there was no significant effect between TAC and sham in female mice for four of these proteins (Fig. 1). Only the abundance of Auh was decreased in female mice subjected to TAC compared with the corresponding sham group. However, it is worth noting that the downregulation of Auh in female mice was not as pronounced as in male mice.

**Table 1:** Data on body and heart weight

	Male		Female	
	Sham	TAC	Sham	TAC
Body weight (g)	27.0 ± 0.6^e^	28.2 ± 0.7^f^	23.4 ± 0.4	23.1 ± 0.2
Heart weight (g)	0.117 ± 0.004	0.206 ± 0.014^c,e^	0.097 ± 0.003	0.154 ± 0.013^b^
Tibia length (mm)	17.3 ± 0.1	17.5 ± 0.2	17.3 ± 0.1	17.1 ± 0.2
Heart weight/tibia length	6.74 ± 0.2	11.83 ± 0.9^c,d^	5.62 ± 0.1	9.04 ± 0.8^a^

Data present mean ± SEM; *n* = 6–10/group

^a^*P* < 0.05

^b^*P* < 0.01

^c^*P* < 0.001 vs. corresponding sham group

^d^*P* < 0.05

^e^*P* < 0.01

^f^*P* < 0.001 vs. female

### Identification of miRNAs putatively regulating the mitochondrial targets

Because miRNAs are important regulators of transcription, we sought to identify those miRNAs that could putatively target the mRNA of the analyzed mitochondrial proteins. In order to do this, we exploited miRNA target prediction algorithms performing a TargetScan analysis [23]. As expected, we identified several putative binding sites for various miRNAs in the mRNA of each of these mitochondrial proteins (Table 2).

**Table 2:** Putative miRNAs regulating the mRNA of mitochondrial proteins

Mitochondrial protein	Putative miRNA regulator
Auh	miR-19b, miR-23a, miR-133a
Crat	miR-23a, miR-24, miR-27b, miR-29a, miR-143, let-7e
Decr1	miR-106a, miR-130a, miR-199b, miR-497a, let-7e
Hadha	miR-19b, miR-27b, miR-130a, miR-143, let-7e
Ndufs4	miR-23a, miR-27b, miR-106a, miR-130a

### Validation of miRNA binding sites in the mRNA of the mitochondrial targets

**Fig. 2:**
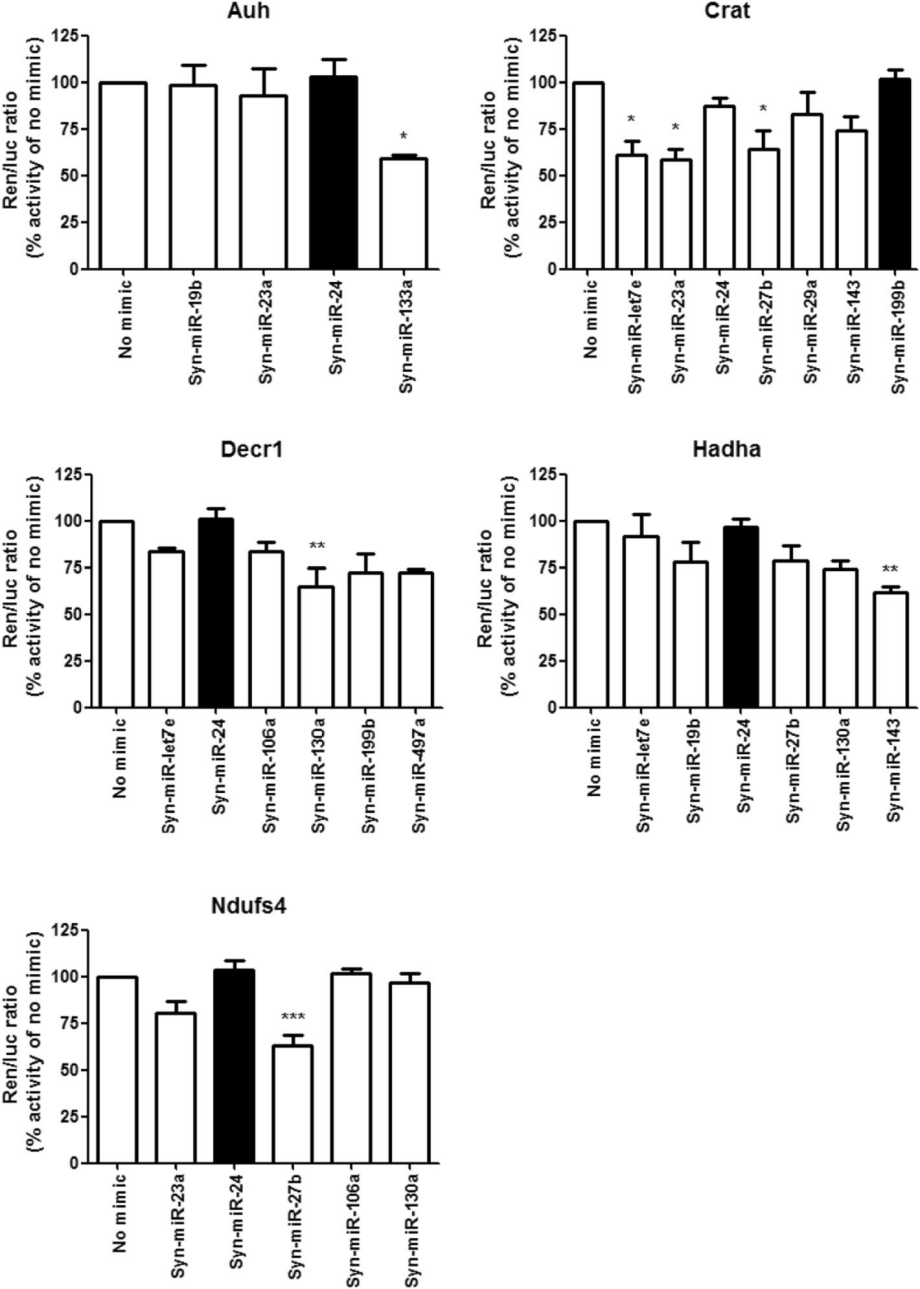
Active miRNA binding sites in the 3′-UTRs of mitochondrial targets. Effect of specific miRNA mimics on complete 3′-UTR clones of Auh, Crat, Decr1, Hadha, and Ndufs4 in cardiac muscle cells (HL-1). Black bars indicate the respective non-specific mimic controls. Data present mean ± SEM. *n* = 5/group; ***P < 0.001, **P < 0.01, *P < 0.05

To confirm the functionality of the binding sites for the identified miRNAs and to examine the possible involvement of these miRNAs in the regulation of the mitochondrial targets, we determined the effect of miRNA mimics in clones carrying the 3′-UTR from each protein-coding mouse mRNA of the mitochondrial targets as part of gain of function experiments in cardiac muscle cells (HL-1). The functional binding of miRNA mimics to the cloned 3′-UTR would lead to reduced expression compared with the no mimic group. To discard false positive findings due to unspecific effects, we employed a miRNA mimic that had no predicted target sites in each corresponding 3′-UTR as negative control (black bars in Fig. 2). As expected, not every putative miRNA binding site was biologically functional (Fig. 2). Only miR-133a reduced Auh expression; miR-23a, miR-27b, and let-7e reduced Crat; miR-130a reduced Decr1; miR-143 reduced Hadha; and miR-27b reduced Ndufs4 (Fig. 2). Taken together, all mRNAs coding for the analyzed mitochondrial proteins carry functional binding sites for at least one of the identified miRNAs.

### Sex-specific miRNA regulation under pressure overload

**Fig. 3:**
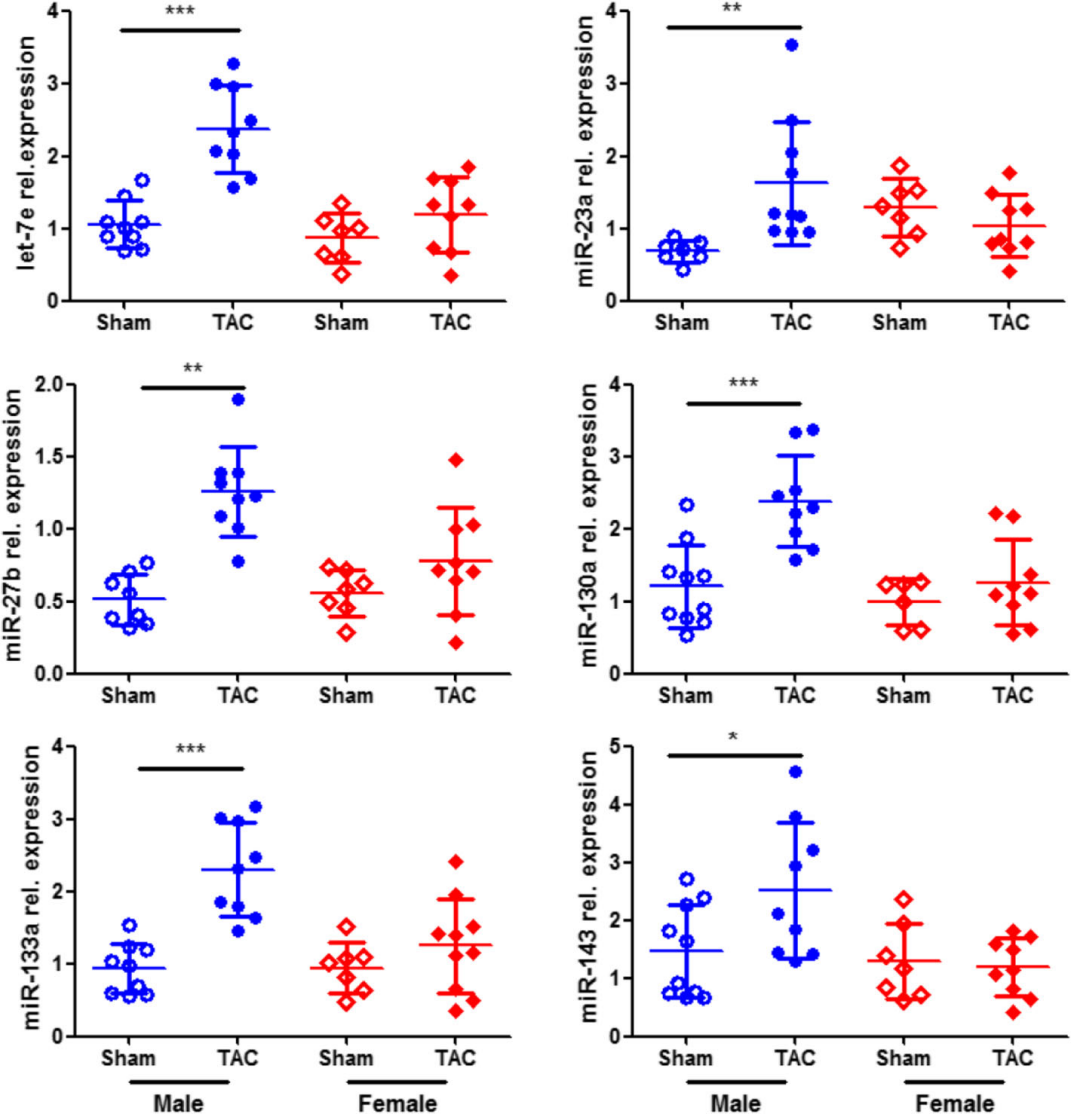
Sex-specific miRNA regulation under pressure overload. The levels of miRNAs were assessed in LV samples from male sham (*n* = 10), male TAC (*n* = 10), female sham (*n* = 7), and female TAC (*n* = 10) mice with intact ERβ. Data are presented in scatter dot plots including mean with SD. ***P < 0.001, **P < 0.01, *P < 0.05

Since the levels of the analyzed mitochondrial proteins were decreased in male mice under PO, we tested the hypothesis that this should be associated with a male-specific induction of the functionally confirmed miRNAs targeting the mRNA of these mitochondrial proteins. On the basis of this, we assessed the levels of these miRNAs and found that their expression was significantly induced in male mice subjected to TAC compared with the corresponding sham group (Fig. 3). In contrast, there was no major effect between TAC and sham in female mice (Fig. 3).

### Involvement of ERβ in miRNA regulation

**Fig. 4:**
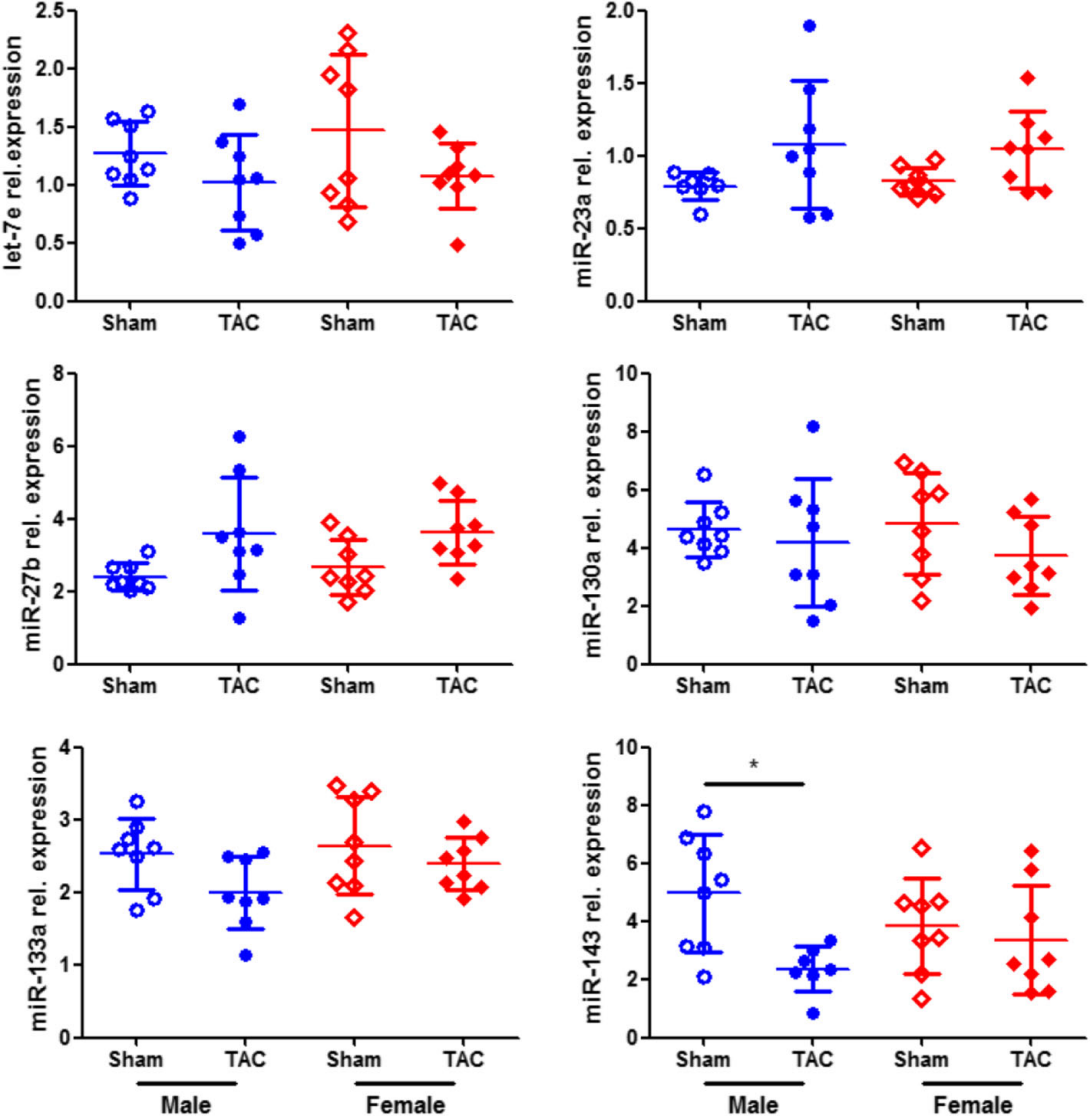
Sex-specific miRNA regulation under pressure overload in ERβ^−/−^ mice. The levels of miRNAs were assessed in LV samples from male sham, male TAC, female sham, and female TAC mice with deficient ERβ (ERβ^−/−^). Data are presented in scatter dot plots including mean with SD. *n* = 8/group; *P < 0.05

The sex-specific miRNA expression together with our previous findings on the effects of ERβ in PO [4, 7, 27] led us to the hypothesis that ERβ may play a role in the regulation of the identified miRNAs. For this purpose, we determined the effect of ERβ genetic deletion (ERβ^−/−^) on cardiac miRNA expression assessing the levels of the miRNAs in LV samples of male and female ERβ^−/−^ mice in response to PO. There was no significant effect between TAC and sham in male or female ERβ^−/−^ mice for five of these miRNAs (Fig. 4). Only the expression of miR-143 was decreased in male mice subjected to TAC compared with the corresponding sham group (Fig. 4).

## Discussion

**Fig. 5:**
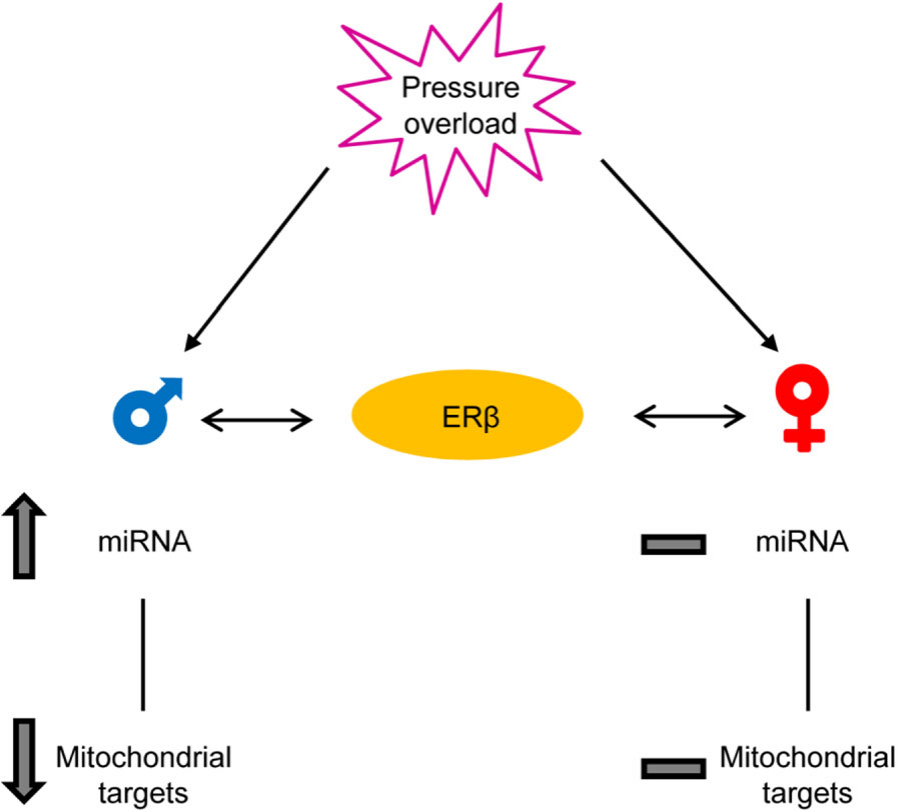
Schematic representation showing the sex-specific effects on miRNA levels and mitochondrial targets in response to pressure overload

In the present study, we found that biological sex plays a major role in the regulation of miRNAs targeting proteins involved in mitochondrial metabolism in the heart, thereby leading to sex-specific cardiac protein regulation (Fig. 5).

There are pronounced differences between males and females in the development of LVH and the transition to heart failure. Although several factors may influence disease progression and outcome [28], the female sex is generally thought to be protective to a certain degree. The effects are prominent at the structural and functional level. Mitochondrial metabolism and bioenergetics in the heart, which is a high energy-demanding organ, are of utmost importance. Based on our previous human and animal studies [4, 7], where we found sex-specific regulation of mitochondrial metabolism-related genes, we report here the significant downregulation of five proteins involved in mitochondrial metabolism in response to PO primarily in male mice. These include Auh, whose aberrant regulation leads to changes in mitochondrial morphology and decreased mitochondrial biogenesis and respiratory function [29]; Crat, Decr1, and Hadha, which play a crucial role in the metabolism and transport of fatty acids for β-oxidation [30–32]; and Ndufs4, an important accessory subunit of the mitochondrial membrane respiratory chain NADH dehydrogenase (complex I) [33]. The overall downregulation of these mitochondrial proteins could have important consequences for sex differences in LVH and male-specific dysfunction documented previously [34, 35], and it requires further research.

Since miRNAs have been identified as novel important regulators of transcription with an ability to simultaneously regulate multiple elements of relevant pathways and several of them have been shown to be regulated in cardiovascular disease [36], including PO [11–15] and cardiac arrhythmias [37], we sought to identify those miRNAs targeting the modulated mitochondrial proteins. Our computational analysis and in vitro experiments identified and validated six miRNAs targeting the mRNAs of the five mitochondrial proteins. Since little is known about the role of sex in the regulation of cardiac miRNAs, we hypothesized and confirmed that these miRNAs are regulated in a sex-specific manner in response to PO. In particular, we found their expression to be induced only in male overloaded hearts. We further ensured that the male-specific downregulation of the target proteins was due to the induction of the regulating miRNAs and not simply due to a general inhibition of mitochondrial proteins in males, since there was no major effect in the levels of Tim23 and Tom40, two important mitochondrial structural proteins involved in the translocation of proteins into the mitochondria. Previous reports corroborate our findings on miR-23a and miR-27b showing induced expression in response to PO [13, 14]. However, others have reported the opposite direction, i.e., downregulation, of miR-133a in response to PO [12, 15], while a third study corroborates our findings on this miRNA [14]. Such discrepancies might be due to differences in the sex, which is not always declared, the strain [38], and age of the mice employed, as well as the time of induction after TAC and its duration before tissue collection [11–15].

The E2/ER axis is expected to be one of the major factors mediating sex-specific effects in the heart. In fact, we have previously reported direct effects of E2 in the heart [19, 38–41], which may differ significantly between the sexes [8, 27, 42, 43]. Notably, we found that E2 exerts deleterious effects in males increasing pro-fibrotic gene expression or impairing contractile function [5, 43]. Along this line, increased circulating E2 levels are a significant predictor of poor prognosis and higher mortality in men with chronic heart failure and reduced ejection fraction [44]. However, the underlying mechanisms and any role of ERβ are incompletely understood. The present data show that in male mice with intact ERβ, there was an increase in miRNA expression in response to PO. In contrast, in male mice with deficient ERβ, there was no significant effect in all miRNAs but miR-143. Overall, these data suggest that ERβ may contribute to a significantly altered expression of cardiac miRNAs.

In addition, the present study raises points that require further research. For example, it is not understood how ERβ might regulate miRNAs in a sex-specific manner. Sex-specific co-factor binding or ER activation may be potential mechanisms involved in this process. Furthermore, miR-143 was downregulated upon genetic deletion of ERβ. This indicates that ERα might also regulate cardiac miRNA expression, which, however, was not within the focus of this study. Similarly, the levels of Auh were decreased in response to PO in female mice, but there was no major effect on miR-133a. This may be due to another miRNA affecting the levels of Auh in females, which, however, was not identified by the present pipeline. To this extent, limitations in the computational analysis for the identification of putative miRNA binding sites are well known in the field. Moreover, due to the previously unrecognized role in the development of PO-induced LVH, the male-specific ERβ-mediated regulation of miRNAs and downstream mitochondrial proteins requires further investigation to identify potential functional consequences on mitochondrial metabolism and contribution to sex-specific cardiac remodeling and function.

## Perspectives and significance

In response to PO, a set of proteins involved in mitochondrial metabolism were regulated in a sex-specific manner, targeted by sex-specific miRNA regulation. A better understanding of the sex differences in the regulation of miRNAs and downstream targets, as well as the elucidation of the underlying mechanisms, will contribute towards the development of more appropriate therapeutic approaches for both sexes.
